# Photoion Mass-Selected Threshold Photoelectron Spectroscopy to Detect Reactive Intermediates in Catalysis: From Instrumentation and Examples to Peculiarities and a Database

**DOI:** 10.1021/acs.jpcc.3c03120

**Published:** 2023-08-21

**Authors:** Patrick Hemberger, Zeyou Pan, Xiangkun Wu, Zihao Zhang, Keisuke Kanayama, Andras Bodi

**Affiliations:** † Paul Scherrer Institute, Villigen 5232, Switzerland; ‡ Institute of Fluid Science, Tohoku University 2-1-1 Katahira, Aoba, Sendai 980-8577, Miyagi, Japan; § Graduate School of Engineering, Tohoku University, 6-6 Aramaki Aza Aoba, Aoba, Sendai 980-8579, Miyagi, Japan

## Abstract

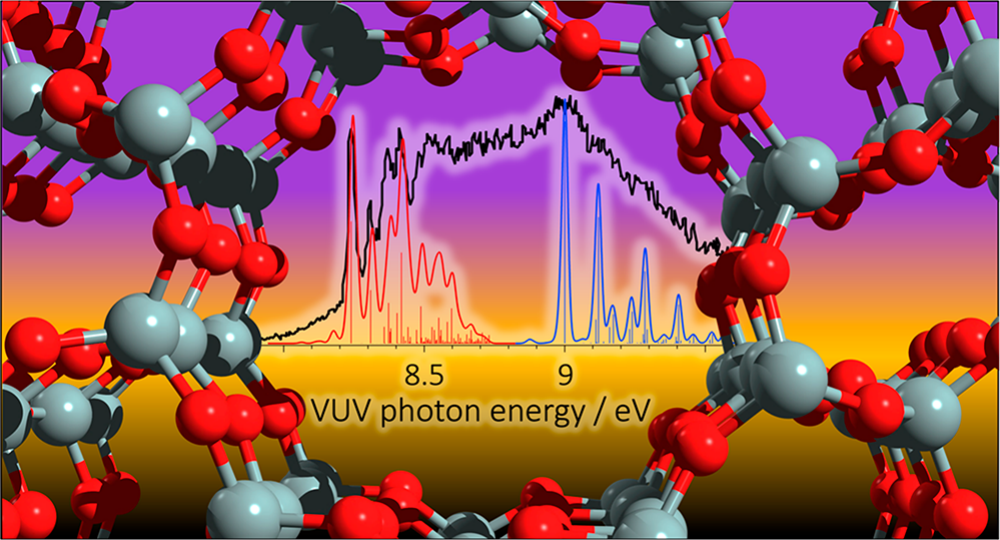

Photoion mass-selected threshold photoelectron spectroscopy (ms-TPES) is a synchrotron-based, universal, sensitive, and multiplexed detection tool applied in the areas of catalysis, combustion, and gas-phase reactions. Isomer-selective vibrational fingerprints in the ms-TPES of stable and reactive intermediates allow for unequivocal assignment of spectral carriers. Case studies are presented on heterogeneous catalysis, revealing the role of ketenes in the methanol-to-olefins process, the catalytic pyrolysis mechanism of lignin model compounds, and the radical chemistry upon C–H activation in oxyhalogenation. These studies demonstrate the potential of ms-TPES as an analytical technique for elucidating complex reaction mechanisms. We examine the robustness of ms-TPES assignments and address sampling effects, especially the temperature dependence of ms-TPES due to rovibrational broadening. Data acquisition approaches and the Stark shift from the extraction field are also considered to arrive at general recommendations. Finally, the PhotoElectron PhotoIon Spectral Compendium (https://pepisco.psi.ch), a spectral database hosted at Paul Scherrer Institute to support assignment, is introduced.

## 1 Introduction

Heterogeneous catalysis accompanies us in our daily lives from the production of plastics, medicine, and fuels to polymer recycling and exhaust gas aftertreatment. Cook-and-look approaches reduce waste and increase selectivities as well as yields to optimize processes economically and ecologically. However, a detailed understanding of the active sites and the reactive intermediates governing the reaction mechanism allows us to improve the process and catalyst design in a more targeted way. Abundant surface species can be identified using IR,^1^ solid-state NMR,^2^ X-ray absorption,^2^ fluorescence,^3^ and photoelectron spectroscopy (PES).^4^ Short-lived intermediates at the heart of catalytic reaction mechanisms hold the key to unveiling the mechanism, but they are more difficult to detect because of their high reactivity and consequently low concentration. If, however, they desorb from the catalyst in the form of radicals, carbenes, or ketenes, they may be identified selectively.^5,6^

Rapid, universal, multiplexed, and sensitive detection tools are needed to capture and assign trace amounts of intermediates. Molecular beam photoionization mass spectrometry (MB-PIMS) extracts samples from catalytic reactors running at up to ambient pressure.^7−9^ The gaseous sample expands into high vacuum, and reactive collisions are efficiently suppressed as a molecular beam is formed. The sample is soft ionized with tunable vacuum ultraviolet (VUV) synchrotron radiation and detected by time-of-flight (TOF) mass spectrometry.^10−14^ By judicious choice of the photon energy to avoid dissociative ionization, peaks can be unambiguously assigned as, e.g., parent peaks of radicals.^7^ Sometimes, when their ionization energies are sufficiently offset, isomers can be distinguished based on their photoionization (PI) spectra. Because of the general absence of sharp features, PI spectra are, however, of limited use if numerous isomers with close-lying ionization energies are present. Valence photoelectron spectra (PES)^15^ often exhibit resolved and isomer-specific vibrational structure, corresponding to transitions from the neutral into the cation according to the Franck–Condon principle. Alas, spectral congestion in species-rich reactive mixtures in catalysis makes PES alone an impractical analytical tool. Photoion mass-selection comes to the rescue by enabling us to match each photoelectron to the cation it originated from in photoelectron photoion coincidence (PEPICO) spectroscopy.^16,17^ By detecting both photoelectrons and -ions in delayed coincidence and kinetic energy analysis of the former, PEPICO yields threshold photoionization mass spectra and photoion mass-selected threshold photoelectron spectra (ms-TPES) as rows and columns of the threshold photoionization matrix. Among synchrotron-based PEPICO experiments,^18−20^ the endstations based at the Swiss Light Source (SLS), Soleil, and the Advanced Light Source (ALS) are the most amenable to ms-TPES detection.^21−23^

In this Perspective, we discuss the advantages, limits, and peculiarities of photoion mass-selected threshold photoelectron spectroscopy as an analytical tool. ms-TPE spectroscopy is a combination of mass spectrometry and photoelectron spectroscopy and identifies elusive intermediates by unveiling vibrational fingerprints in each *m*/*z* channel when probing reactive mixtures. These spectroscopic features correspond to vibrational excitation of the cation and can be isomer-selectively assigned to the neutral species, similar to infrared spectroscopy. We discuss the instrumentation from the molecular beam interface to spectrometer design and capabilities. We briefly explain the quantum chemical selection rules that are crucial for the sensitivity and selectivity of photoionization techniques. A few of the most recent heterogeneous catalysis examples are detailed, which shed new light on reaction mechanisms by the detection of reactive ketenes and radicals. Furthermore, we discuss peculiarities, insights, and limitations of the technique, namely, the temperature and field dependence of the spectra. Finally, we introduce the **P**hoto**E**lectron **P**hoto**I**on **S**pectral **CO**mpendium (PEPISCO) https://pepisco.psi.ch, an open database for (photoion mass-selected threshold) photoelectron and photoionization spectra, hosted at Paul Scherrer Institute.

## 2 Instrumentation and Methodology

### 2.1 PEPICO Instrumentation for ms-TPE Spectroscopy

**Figure 1:**
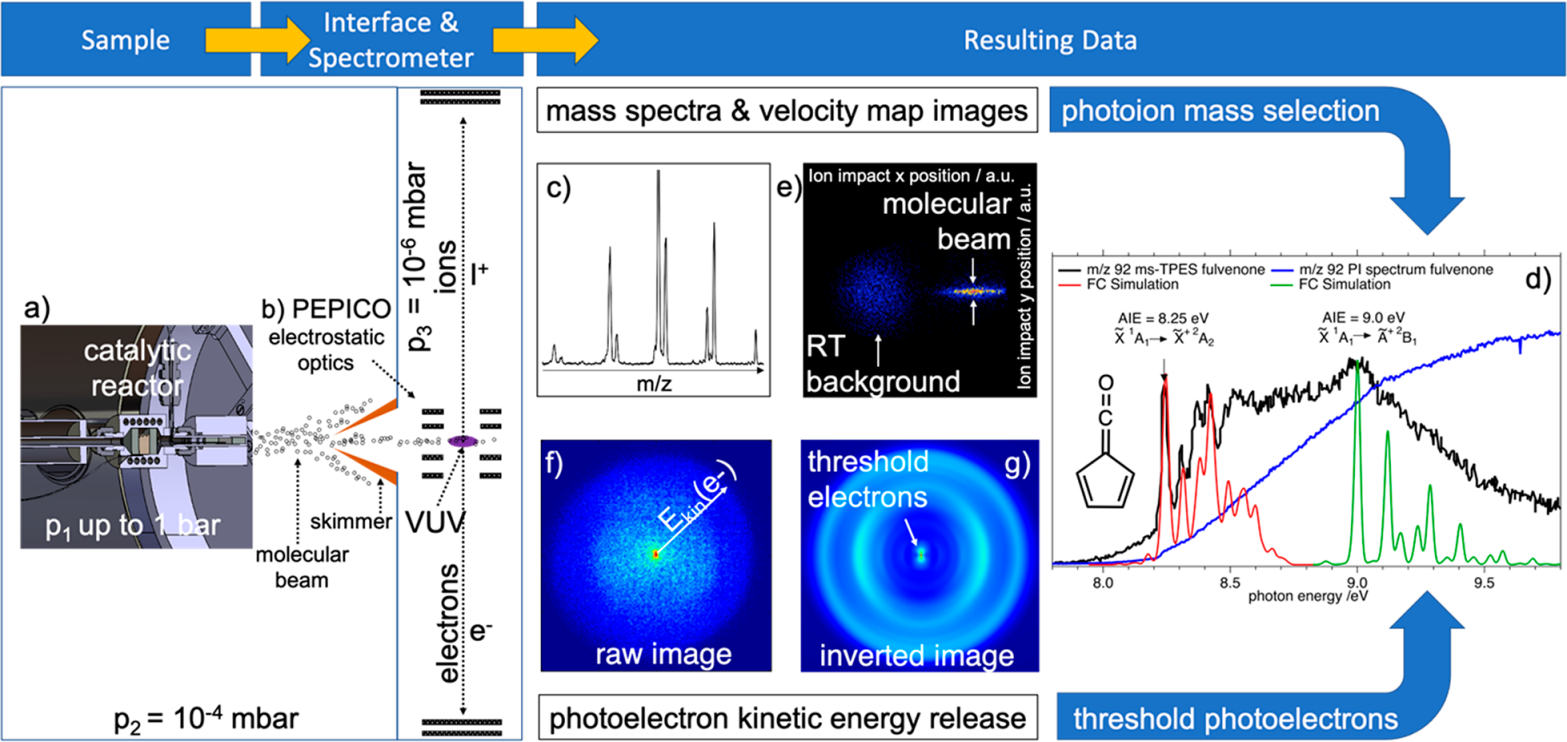
*Operando* photoelectron photoion coincidence setup at the SLS. The sample emanates from a catalytic reactor (a) from up to ambient pressure, *p*_1_, forms a molecular beam in the source chamber, and is skimmed as it enters the high-vacuum ionization chamber with the PEPICO optics (b). Photoions detected in delayed coincidence with all electrons yield the mass spectrum (c), 1st analytical dimension. Ion velocity map images (e, 2nd analytical dimension) identify sample provenance and kinetic energy release upon dissociative ionization. Photoelectron velocity map imaging (f) yields photoelectron spectra at fixed photon energy by VMI reconstruction (g) and photoion mass-selected threshold photoelectron spectra (d) by hot electron subtraction and photon energy scanning (3rd analytical dimension). Reprinted (adapted) with permission from ref.^24^ Copyright 2017 American Chemical Society. Reprinted (adapted) with permission from ref.^25^ Copyright 2020 Wiley.

Photoion mass-selected threshold photoelectron spectroscopy requires the detection and analysis of electrons and ions in delayed coincidence. In *operando* photoelectron photoion coincidence spectroscopy, a catalytic reactor is coupled via a differentially pumped sampling interface to a spectrometer, as depicted in Figure 1a.^21−23,26^ The gaseous sample leaving the heated reactor expands into high vacuum (*p*_2_ ≈ 10^–4^ mbar), forming a molecular beam, in which collisions are suppressed and reactive intermediates conserved. Depending on the expansion conditions, the beam may be supersonic, is skimmed and enters the ionization chamber at *p*_3_ ≈ 10^–6^ mbar. The beam intersects with the monochromatized vacuum ultraviolet (VUV) synchrotron light in the ionization volume, where a photoelectron and a photoion are produced in each ionization event (Figure 1b). Due to quasi-continuous synchrotron radiation, the ionization rate is constant. Furthermore, thanks to the low sample density and the short (<20 μs) ion time-of-flight, concurrent ionization events are rare. Therefore, when electrons and ions are detected in delayed coincidence, they almost always belong to the same ionization event. The nature of the false coincidence background in high count-rate experiments is also well understood^27^ and can be addressed.^22,28^ The electron detection is virtually instantaneous after ionization, which means that the delay at which the cation is detected corresponds to the ion time-of-flight, hence, the *m*/*z* ratio. Thus, the electron and ion detection time differences yield a photoionization mass spectrum (PIMS) as depicted in Figure 1c. On the one hand, mass spectra at different photon energies and reaction conditions (temperature, pressure, concentrations, or catalyst) often enable the identification of especially the light constituents of the effluent and provide valuable insights into the mechanism. By scanning the photon energy and plotting the intensity of an *m*/*z* channel, photoionization spectra (PIS) can be obtained (1st analytical dimension; see Figure 1d, right, blue line). The isomer-specificity of PIMS is, however, limited because the PIS generally exhibits only broad and unstructured features.

Velocity map imaging (VMI) of the ions using fast position sensitive detectors, the second analytical dimension in double imaging *i*^2^PEPICO spectroscopy, yields momentum information, which distinguishes the molecular beam signal from the scattered and thermalized background signal in the ionization chamber (Figure 1e). Accounting for sampling effects is particularly important when quantifying the signal of reactive intermediates, which may not survive wall collisions.^6,29^ Furthermore, dissociative ionization is associated with kinetic energy release (KER). As the molecular beam signal is skimmed and has a narrow lateral velocity distribution, KER shows up unmistakably in the beam signal, indicating that the *m*/*z* signal is the result of the fragmentation of a heavier species. Photoelectrons are also velocity map imaged. When electrons in coincidence with a cation of a certain *m*/*z* are discriminated for (Figure 1f), inverting the electron VMI at a fixed photon energy^30^ gives rise to photoion mass-selected photoelectron spectrum (ms-PES, Figure 1g). Although this approach is best suited to address angular anisotropies, e.g., in photoelectron circular dichroism experiments,^31^ VMI energy resolution decays with increasing energy, and fast electrons are not resolved as well as slow ones. If, however, only close-to-zero kinetic energy electrons are taken into account, photoion mass-selected threshold photoelectron spectra (ms-TPES) can be obtained with high energy resolution (Figure 1d). Threshold electrons are imaged onto the center spot on the detector. Kinetic electrons with no prompt lateral momentum also contribute to the center signal, and the signal in a ring area around the center spot can be used to approximate the resulting hot electron contamination.^32^ Thus, the ms-TPES can be obtained by a simple subtraction scheme as shown by Sztáray and Baer.^32^ Alternatively, the photon energy is scanned, and only the well-resolved slow electrons are relied on to plot slow photoelectron spectra after the reconstruction of multiple photoion mass-selected electron velocity map images in slow photoelectron spectroscopy (ms-SPES, see Section 4.2 below).^33^ Thus, electron imaging adds a third analytical dimension to imaging photoelectron photoion coincidence detection because, in contrast to the PIS, ms-TPES and ms-SPES often exhibit clearly resolved, isomer-specific vibrational progressions and distinct peaks (Figure 1d, black lines). To show how to interpret the spectra routinely even in the absence of reference data, we briefly review how Franck–Condon factors govern the intensity of vibrational transitions in the photoelectron spectrum.

### 2.2 Threshold Photoelectron Spectra and Selection Rules

**Figure 2:**
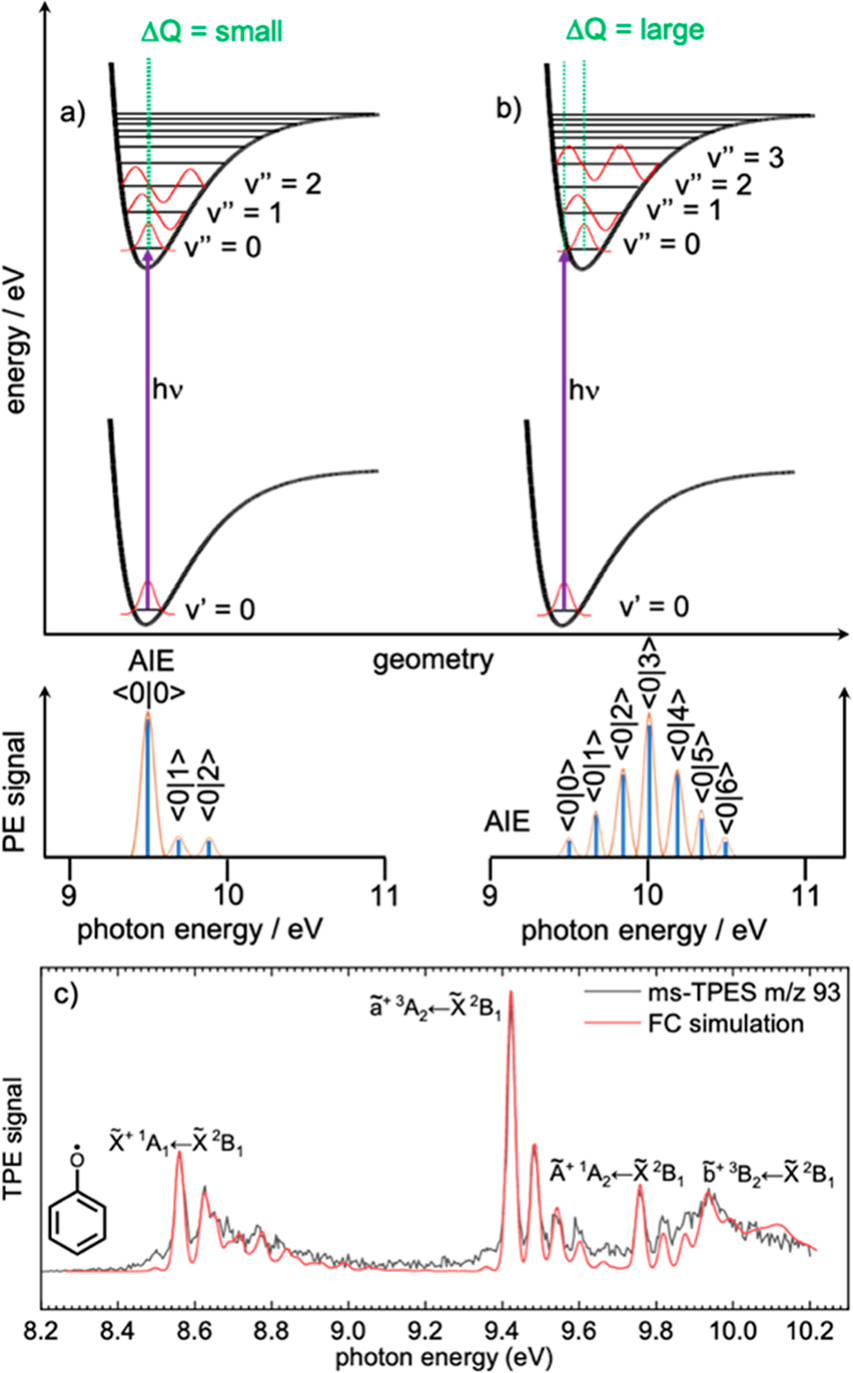
(a, b) Franck–Condon principle: the vibrational wave function overlap determines the vibrational structure in the photoelectron spectrum. (c) Comparison between simulation and experiment of the phenoxy radical. Reprinted (adapted) with permission from ref.^36^ Copyright 2022 American Chemical Society.

Valence photoionization corresponds to the removal of a valence electron from a species, which, in Koopmans’ approximation,^34^ corresponds to the removal of an electron from a valence molecular orbital. The transition probability *P*_if_ for an ionization transition between an initial state (*i*, neutral) and a final state (*f*, ion) can be derived from solving the time- and position-dependent Schrödinger equation, which, after the separation of nuclear (*R*) and electronic (*R_e_*) coordinates, yields the following expression in the Born–Oppenheimer approximation^35^
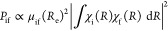
where *μ*_if_(*R*_e_) corresponds to the transition dipole moment of the electronic wave function at the equilibrium geometry. The second term, i.e., the nuclear wave function overlap integral, also called the Franck–Condon factor (FCF), gives the intensity of the vibrational transitions within the band belonging to a certain final cation electronic state. The transition dipole moment *μ*_if_(*R*_e_) is nonzero in ionizing transitions, and the removal of an electron is generally allowed. This explains the universality of ionization detection. Figure 2 shows two cases for a diatomic molecule and the resulting vibrational progression. If the change in the equilibrium geometry (Δ*Q*) from the neutral into the cation is small, the overlap integral is largest between the ground-state neutral and ion-state vibrational wave functions ⟨0 | 0⟩, leading to a high FC factor for the intense fundamental transition (Figure 2a).

Stepping up in the vibrational energy of the cation leads to monotonously decreasing FCFs for the ⟨0 | 1⟩, ⟨0 | 2⟩, etc. transitions and, thus, lower intensity peaks in the band. The difference between the ⟨0 | 0⟩ and ⟨0 | 1⟩ peak positions in the experimental spectrum is equal to the vibrational frequency of the FC active mode in the cation. The second case, depicted in Figure 2b as large Δ*Q*, shows large geometry change upon ionization, which results in a longer equilibrium bond distance in the cation than in the neutral. As a result, the FCF for the fundamental transition ⟨0 | 0⟩ is small. In some cases, such as in the ground-state band of the dichloromethane photoelectron spectrum, this may lead to the fundamental transition not even being visible in the spectrum.^41^ With increasing internal energy of the cation, the vibrational overlap integral also increases at first and has a maximum at ⟨0 | 3⟩. The intensities decrease from there on, which results in the vibrational progression shown in Figure 2b. Since the experimental spectra deviate from the harmonic approximation, photoelectron spectroscopy also allows the determination of the anharmonicity constant, in favorable cases.

How does the Franck–Condon envelope help identify reactive species in complex catalytic reaction mixtures? Isomers of polyatomic molecules often possess different adiabatic ionization energies (AIEs), offset excited-state bands in the photoelectron spectrum, and may undergo different geometry change upon ionization, which results in a difference in the FC active modes and the resulting vibrational fine structure. This can be used as an isomer-specific fingerprint. FCF calculations in the double harmonic approximation have been implemented in numerous quantum chemical packages, such as ezFCF (eZspectrum),^35^ Gaussian 16,^42^ Molpro,^43^ and FCFit.^44^ In the absence of active large-amplitude motions, i.e., internal rotations,^45^ the double harmonic approximation reproduces the vibrational fine structure generally well. As an example, the phenoxy radical ms-TPES is depicted in Figure 2c and shows transitions into singlet and triplet ground and excited states, respectively, reproduced by FC spectral modeling.^36^ There are, however, instances, when the spectral fine structure is not entirely or not at all reproduced by a double harmonic Franck–Condon simulation. (Pseudo-)Jahn–Teller distortions or large-amplitude motions that are active upon ionization can require one to go beyond the harmonic approximation to successfully model the ms-TPE spectra still within the Born–Oppenheimer approximation.^46−48^ Furthermore, the ms-TPE spectrum of fulvenone ketene (Figure 1d) is affected by lifetime broadening due to close-lying electronic states, which are strongly coupled by a conical intersection.^25^ This washes out the vibrational fine structure of the higher-lying state, which the FC model cannot predict. Thus, both reference photoelectron spectra and Franck–Condon simulations can contribute to ms-TPES species assignment in reaction mixtures, which goes beyond the possibilities of PI mass spectral speciation.

## 3 Applications

In the following, we highlight recent investigations in which the isobar- and isomer-selective capabilities of ms-TPES detection contributed to elucidating the reaction mechanism.

### 3.1 Lignin Catalytic Fast Pyrolysis

**Figure 3:**
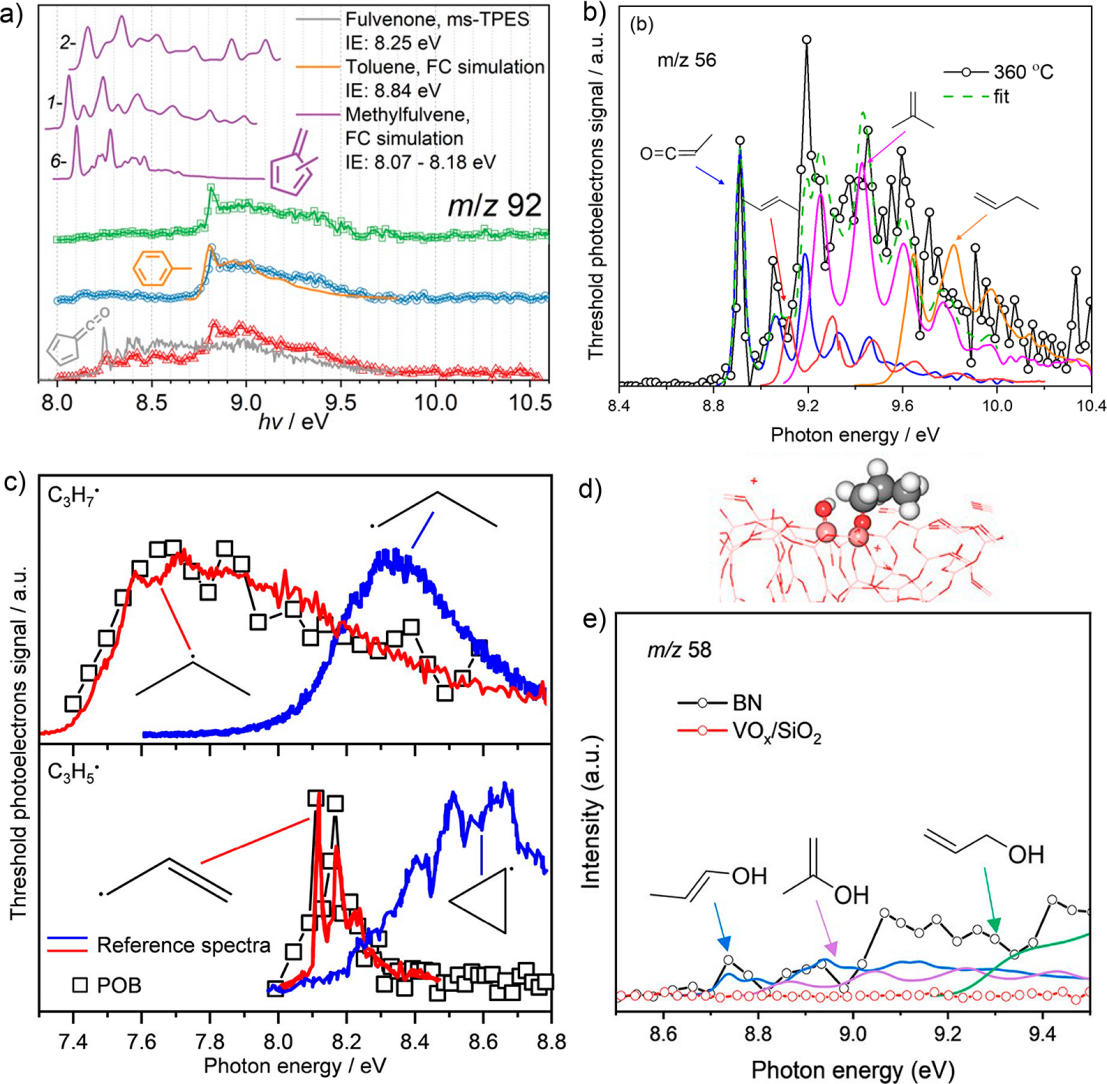
Threshold photoelectron spectra of (a) fulvenone, toluene, and methylfulvene as well as the *m*/*z* 92 ms-TPES in the CFP of 2-methoxyphenol (2-MP, red), 3-MP (blue), and 4-MP (green), (b) methylketene detection in MTO and (c) *i*-propyl and allyl radicals in propane oxybromination together with reference spectra. (d) Strongly bound *n*-C_3_H_7_ on the >BO-OB< site of BN. (e) ms-TPES detection of ethenol. (a) Reprinted (adapted) with permission from ref.^37^ Copyright 2022 Royal Chemical Society. (b) Reprinted (adapted) with permission from ref.^38^ Copyright 2022 Wiley. (c) Reprinted (adapted) with permission from ref.^39^ Copyright 2020 American Chemical Society. (d,e) Reprinted (adapted) with permission from ref.^40^ Copyright 2023 American Chemical Society.

Toluene is a prevalent product in the catalytic fast pyrolysis (CFP) of lignin constituents. The elusive fulvenone ketene (c-C_5_H_4_=C=O) cannot be detected by gas chromatography-mass spectrometry (GC/MS) analysis, but it has the same nominal mass, *m*/*z* 92.^37,49,50^ The ms-TPES spectrum in Figure 3a shows contributions of toluene (features above 8.8 eV) in the catalytic pyrolysis of all three methoxyphenol (MP) isomers over H-ZSM5. Fulvenone, on the other hand, shows distinct vibrational features at 8.25 eV and appears only in the CFP of 2-MP (guaiacol, red curve).^25^ Due to the high reactivity of fulvenone toward phenol and cyclopentadiene (R1), the conversion of the *ortho* isomer (2-MP, guaiacol) is much higher than that of 3- and 4-MP. Thus, ms-TPES detection can unveil isomer-specific reaction mechanisms by revealing the presence or absence of reactive intermediates as additional analytical dimension in photoionization measurements.
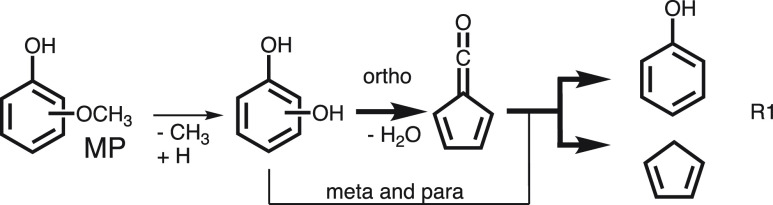


### 3.2 Initiation of Methanol-to-Olefins Process

Ketenes have also been identified in the methanol-to-olefin (MTO) process over zeolites recently. Previously, ketenes were hypothesized to be only early intermediates in the initiation phase. However, they have now been shown to be responsible for the formation of the first olefins.^51,52^ To boost ketene formation, we used methyl acetate as reactant (R2, in Figure 3b), and high yields of H_2_C=C=O were already obtained at 260 °C reactor temperature over HZSM-5.^38^ When the temperature was raised to 360 °C, methylketene (CH_3_(H)C=C=O) could also be observed, as depicted in Figure 3b. The methylketene signal is clearly distinguishable from the isobaric butene peaks because of its distinct fundamental transition at the AIE at 8.85 eV and its vibrational fingerprint thereafter. Due to the close-lying ionization energies of methylketene and 2-butene ms-TPES detection is clearly advantageous in comparison to photoionization mass spectrometry. Mechanistically, methylketene is produced via methylation of ketene according to R2 over zeolite (Ze) and decarbonylates rapidly to form the first olefin, ethene. This confirms the theoretically hypothesized ketene-to-olefin route and solves a long-standing conundrum about the MTO mechanism. Ketenes are currently in the spotlight as central reactive intermediates^53,54^ not only over zeolite-catalyzed MTO process but also during syngas-to-olefin reactions or in the boron nitride catalyzed oxidative dehydrogenation of propane (see chapter 3.4).^40^ In addition, formaldehyde is responsible for catalyst deactivation but can also increase the selectivity toward aromatics. PEPICO detection of this reactive species is currently also of great interest in the MTO community.^55,56^
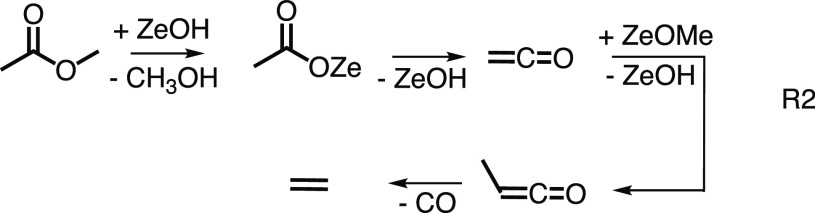


### 3.3 Radical Pathways during Alkane Activation

Another example concerns C–H activation in propane oxybromination to yield propylene. In the initial step, HBr is catalytically oxidized on the CrPO_4_ catalyst surface and released in the gas phase as Br· radicals.^39^ Br· abstracts hydrogen from abundant propane in a gas-phase reaction. Operando PEPICO spectroscopy enabled the measurement of the ms-TPE spectra of numerous intermediates with unprecedented selectivity. It was found that the thermodynamically more stable *i*-propyl radicals (Figure 3c) were solely produced, while *n*-propyl radicals were absent in the gas phase. In addition, a sequence of lighter C_3_ radicals could also be identified. A subsequent bromination, dehydrobromination, and H-abstraction (see R3 in Figure 3) reaction yields allyl radicals (C_3_H_5_, *m*/*z* 41), while the cyclopropyl isomer could not be observed in the reaction mixture. The adiabatic ionization energies and photoelectron spectra of the two isomers are significantly different, making it easy to tell them apart. Furthermore, observation of propargyl (C_3_H_3_, *m*/*z* 39) and the C_6_H_6_ isomers fulvene (c-C_5_H_4_=CH_2_) and benzene (R3) shed new light on the side reactions representing the initial stages of polycyclic aromatic hydrocarbon formation, which finally leads to coke and, thereby, catalyst deactivation. Upon exchanging bromine to chlorine in oxychlorination, gas-phase intermediates became absent, as the reaction is surface-confined and the selectivity to propylene increases.^39^ Thus, operando PEPICO detection allowed us to distinguish between gas-phase and surface-confined mechanisms.
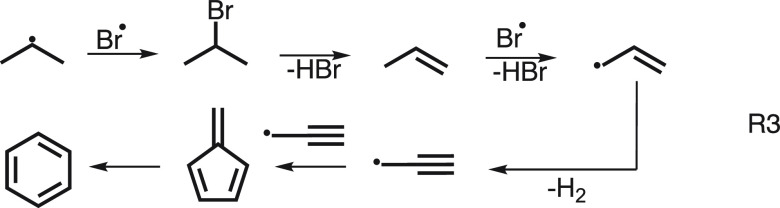


### 3.4 Radical- and Oxygenate-Driven Routes in Propane Oxidative Dehydrogenation

Propane oxidative dehydrogenation (ODHP) has been widely performed using vanadia catalysts (VO_*x*_). However, this process suffers from overoxidation into thermodynamically more stable CO and CO_2_. Boron nitride (BN), on the other hand, is less prone to overoxidation and selectively produces propylene.^40^ The transformation of propane to propylene follows a surface-confined as well as a gas-phase route. In the former, propane is strongly bound as *n*-C_3_H_7_ to the >BO–OB< sites and desorbs only as C_3_H_6_ after hydrogen transfer to the catalyst (see Figure 3d). In contrast, if the reaction occurs on the >BO dangling site, propane is activated similarly, but propylene may react further to allyl, which then desorbs in the gas phase according to R4.
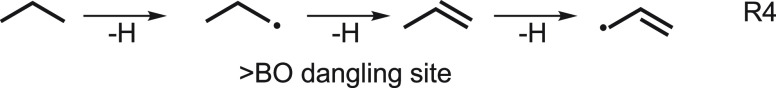


In addition to allyl radicals, we also detected methyl radicals using ms-TPE spectroscopy, while ethyl and propyl were absent due to rapid dehydrogenation to ethylene and propylene. Methyl and allyl radicals can be hydrogenated and explain the observation of methane and propylene in the gas-phase H-acceptor route.
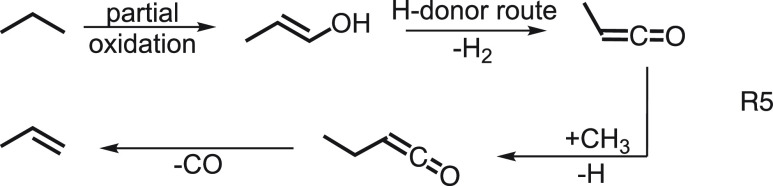


In contrast to the vanadia catalyst, overoxidation is prevented by partial oxidation to propenols (see Figure 3e) and to ketenes (H-donor route), which after methylation and decarbonylation also yield propylene (R5). Thanks to the ms-TPES detection of ketenes, ethenols, and radicals, the existence of a gas-phase reaction mechanism could be unveiled. The low desorption energy of reactive species from the >BO dangling sites is the key to prevent overoxidation to CO_2_ over BN catalysts.

## 4 Peculiarities of Threshold Photoelectron Spectroscopy

We have discussed the advantages of photoion mass-selected threshold photoelectron spectroscopy. However, as with every technique, data acquisition and analysis approaches, sampling conditions, and the question of reference data sets must also be addressed to fully exploit the analytical prowess of ms-TPES detection. In the following, we discuss the robustness of ms-TPES detection. How sensitive are ms-TPE spectra toward sample temperature? How does the strength of the electrostatic field, extracting both the ions and electrons from the ionization volume, affect the spectrum? Are the spectra sensitive to different data analysis techniques?

### 4.1 Temperature Dependence

**Figure 4:**
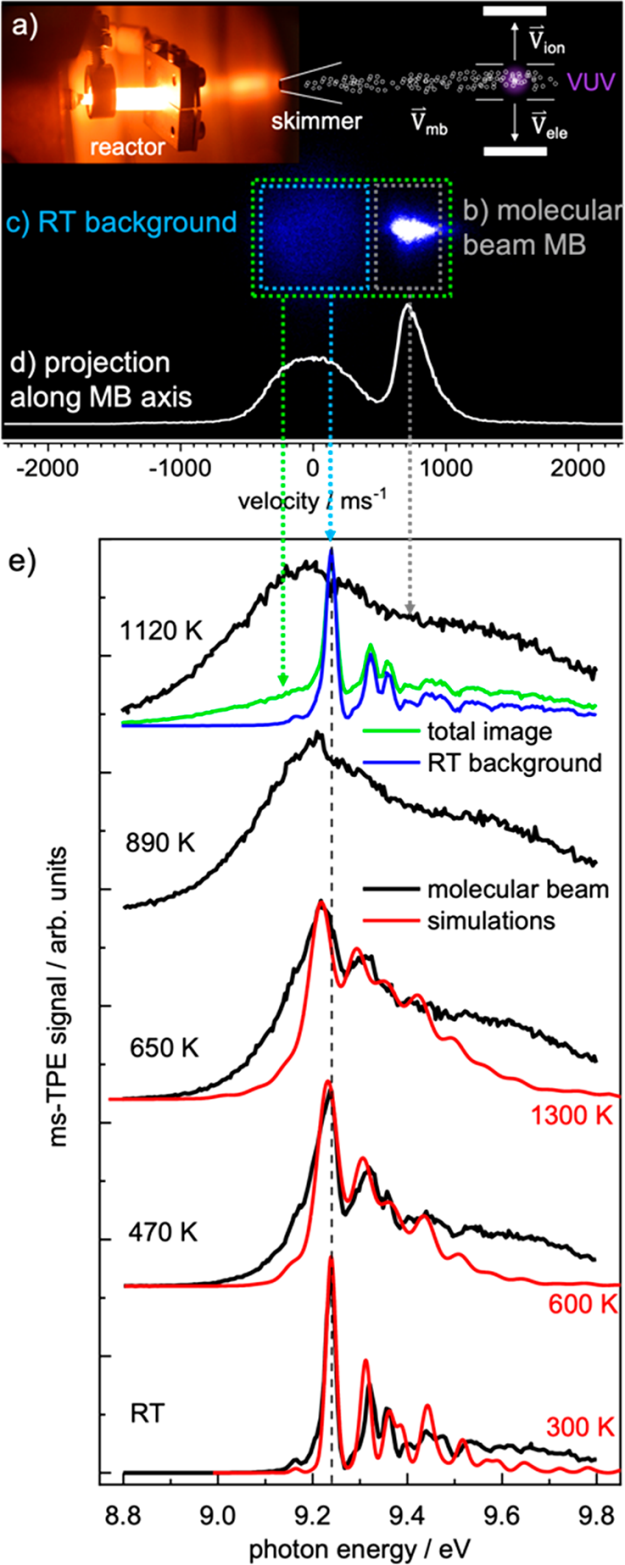
(a) Pyrolysis microreactor with schematic representation of the molecular beam (MB) and ionization region. (b) Ion velocity map imaging distinguishes the gas jet leaving the reactor directly (MB) from the scattered, rethermalized (RT) signal (c). (d) Velocity distribution along the MB axis. (e) Temperature-dependent benzene ms-TPES. The MB (black spectrum), RT (blue spectrum), and total signal (green) are shown for comparison. Reprinted (adapted) with permission from ref.^29^ Copyright 2022 American Chemical Society

Reaction temperatures in catalysis reactors and microreactors may reach several hundred to thousand °C. The expansion in a vacuum often takes place from a pressure of only a few hundred mbar. At such sampling conditions, collisional cooling in the molecular beam expansion only effectively cools the translational degrees of freedom, which leads to a neutral sample with an essentially unchanged internal energy distribution.^29^ Hot and sequence band transitions can be modeled within the Franck–Condon approximation, but the increased rotational broadening impedes the isomer-selective assignment of stable and reactive intermediates in catalysis experiments, especially if hot reactors are used. Furthermore, the spectral broadening of certain, notably aromatic, samples goes beyond the thermal effect predicted by the Franck–Condon approximation, as shown for benzene in Figure 4.

Ion velocity map imaging (Figure 4a) can overcome this challenge.^21−23^ VMI disperses the signal in the detector plane according to the velocity distribution of the neutral. The molecular beam signal (Figure 4b, gray box) exhibits high velocities with a narrow distribution along and low velocities perpendicular to the expansion axis (Figure 4d), thanks to translational cooling and skimming, respectively. The neutral scattered background (Figure 4c, blue box), on the other hand, results from collisions with the chamber walls and can be distinguished spatially from the molecular beam (Figure 4b, gray box).

Thanks to the coincident detection, we can plot three sets of ms-TPES at a single reactor temperature (e.g., 1120 K, Figure 4e). (i) The total ion image (green spectrum, Figure 4e) shows a strong hot band contribution below the AIE of benzene and a signal offset at higher photon energies. This is in contrast (ii) to the RT background VMI selected spectrum (Figure 4e, spectrum in blue), exhibiting almost baseline-resolved vibrational transitions. The difference between the two spectra (iii) is evidenced in the molecular beam ms-TPE spectra (MB selection in Figure 4e) showing significant hot and sequence band contributions. By looking only at the MB VMI selection (see black ms-TPE spectra from RT to 1120 K in Figure 4e) a clear increase of the hot bands with increasing reactor temperature is evident, which is more than that predicted by the Franck–Condon approximation (red curve). In favorable cases, such as for the allyl radical,^29^ the broadening of the spectrum is indeed predicted by the FC model accurately. However, even this lowers the dynamic range of the experiment and complicates the identification of different isomers with low mole fractions, which is otherwise often central to establishing branching ratios of bimolecular association reactions.^57^ This broadening can be resolved by integrating exclusively the RT background signal (blue curve in Figure 4e), which remains virtually indistinguishable from the room-temperature spectrum of the reactor (lower trace) even at the 1120 K reactor temperature. Thus, wall collisions result in room-temperature sample, suppressing hot- and sequence-band transitions and the rotational broadening. On the one hand, most volatile molecules including resonantly stabilized radicals, such as allyl and phenoxy,^36^ survive wall collisions making RT ms-TPES easy to record with the help of cation VMI. On the other hand, involatile species may be adsorbed on the chamber walls and may only slowly desorb, resulting at best in a loss of time resolution. Furthermore, as shown in the case of the hydroxyl radical,^58^ reactive intermediates may not survive wall collisions and elude detection as rethermalized RT cooled signal. Based on these insights derived from the ion velocity map images, we arrive at the following guiding principles: Quantification by ms-TPES or photoionization mass spectrometry (PIMS) must be carried out using the hot molecular beam (MB) signal unless there is experimental evidence for the species’ survival of wall collisions with the vacuum chamber. Furthermore, reactions yielding nonvolatile species, such as molecular iodine during the pyrolysis of allyl iodide, can also lead to concentration/selectivity errors in the scattered background signal, thus changing the apparent kinetics.^29^ On the contrary, isomer-selective identification can be enhanced by relying exclusively on the ms-TPE spectrum associated with the scattered, room-temperature background ion signal. The higher resolution of the spectra makes the overall fit with FC modeled or reference spectra much more reliable and conclusive.

### 4.2 Field- and Kinetic Energy Dependence

On the one hand, the kinetic energy analysis of the electron velocity map images is primarily the experimenter’s choice. The dependence of the photoelectron spectrum on the extraction field is, on the other hand, determined by the Stark effect: higher extraction fields lead to field ionization of lower-energy high-*n* Rydberg states, which shifts the onset of the TPES to red (lower photon energies). Since both effects are coupled and can contribute to a shift and a broadening of the ionization transitions, the isomer-sensitivity in a catalytic experiment may be affected. Thus, we address these effects briefly together.

As discussed by Chupka, the Stark shift is smaller in the diabatic limit, applicable in pulsed experiments, and
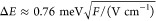
in the adiabatic limit, where *F* is the constant extraction field.^59^ This was confirmed in constant extraction field PEPICO experiments^60^ by TPES measurements on CH_3_I, Ar, and N_2_ and yields a red shift of the ionization onset of ca. 10 meV at the routinely used 200 V cm^–1^ extraction field. The peak maximum of the origin transition corresponding to the adiabatic ionization energy has sometimes been assumed to be shifted by this amount, too.^61^ However, the TPES peak positions were found to be insensitive to the extraction field in certain halogenated hydrocarbons.^62^ These threshold photoelectron spectra were plotted with the help of hot (kinetic energy) electron subtraction as proposed by Sztáray and Baer.^32^ Here, a small ring area around the center (see Figure 1g), threshold electron spot, is assumed to be representative of the hot electron contamination of the center signal in the velocity map image, i.e., of kinetic electrons without off-axis momentum imaged to the center spot. Especially during low-signal experiments, it is worthwhile to increase the center spot size, thereby trading electron kinetic energy resolution for more signal. This, however, might affect the isomer-resolving capabilities of ms-TPES.

Hot electron subtraction is normally based on a center area with a 0.8–1.0 mm radius on the detector. This also means that the cutoff energy for threshold electrons is a function of the extraction field and corresponds to 4 meV at 220 V cm^–1^.

On the one hand, the spectrum can be expected to broaden toward higher photon energies when the extraction field strength is increased while keeping the center radius constant, as faster electrons will be accepted as threshold. On the other hand, higher extraction fields also lead to a higher Stark shift, moving the rising edge of the peak to lower photon energies.

To address these two effects, we have recorded benzene, acetonitrile, and dichloromethane threshold photoelectron spectra using 22–220 V cm^–1^ extraction field and plotted the spectrum using various kinetic energy cutoffs to gain an overview of the benefits, drawbacks, and pitfalls of TPES acquisition.

**Figure 5:**
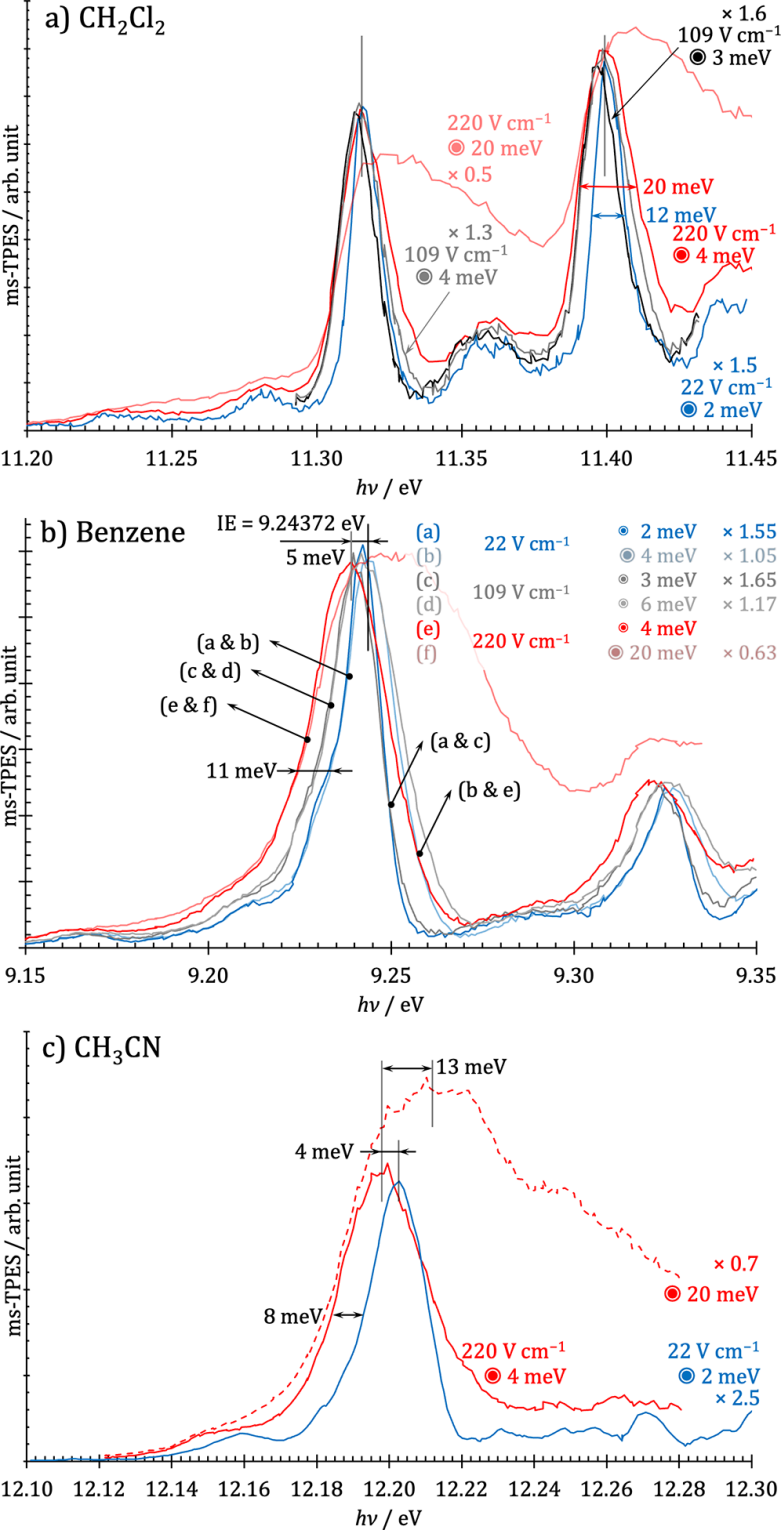
Comparison of the (a) dichloromethane, (b) benzene, and (c) acetonitrile ms-TPE spectra as a function of the extraction field (in V cm^–1^) and the kinetic energy cutoff (in meV) in the hot electron subtraction.

In the case of dichloromethane (Figure 5a), the two effects cancel each other out, and the peak only broadens, but its maximum barely shifts as the extraction field is increased. At 220 V cm^–1^ and a 20 meV kinetic energy cutoff, the peak maximum stays, but broadening toward the blue shoulder is apparent. In case of benzene (Figure 5b), however, the red shift of the rising edge, brought about by the Stark shift, is about twice as large as the blue shift of the falling edge, meaning that the peak maximum red shifts by about half the Stark shift and is found at 9.239 eV at an extraction field of 220 V cm^–1^ instead of the known ionization energy of 9.244 eV.^63^ The same effect was found in acetonitrile (Figure 5c), as well, suggesting that subtracted TPES maxima of organic compounds are likely to shift to lower energies by ca. half of the Stark shift. To the best of our knowledge, all reported ionization energies obtained by hot electron subtraction either disregard the Stark shift or consider it in its entirety, meaning that the ionization energies are likely too low or too high by 3–5 meV, respectively. The reason TPES peak maxima are invariant of the extraction field in halogenated compounds could be the rising electron yield with electron kinetic energies, leading to a more significant blue shift of the falling edge of the peak as the field and the electron energy cutoff are increased. This is also supported by the Stark peak broadening from a full width at half-maximum of 30 to ca. 100 meV upon raising the electron cutoff energy from 4 to 20 meV, corresponding to a center radius of 0.8 and 8 mm, respectively, at a 220 V cm^–1^ extraction field. Thus, raising the electron cutoff energy above 10 meV leads to disproportionate peak broadening and should be avoided when plotting hot electron subtracted TPES.

When compared with usual, ca. 10 meV step size, determined by the difficult balancing act between achieving a suitable signal-to-noise ratio during the finite measurement time while resolving the vibrational fine structure of the ms-TPES completely, the effect of the threshold cutoff energy within 10 meV and the Stark shift induced by an extraction field below 300 V cm^–1^ appears to be minor. In conclusion, the field and kinetic energy dependence effects may only play a role when ms-TPES detection is pushed to the limit considering constitutional or conformational isomers with barely differing ionization energies. Otherwise, it is a robust analytical tool and is not sensitive to the particularities of data acquisition.

### 4.3 SPES vs TPES

Alternative data analysis strategies exist when electron VMI is used, besides the hot-electron subtraction introduced by Sztáray and Baer.^32^

**Figure 6:**
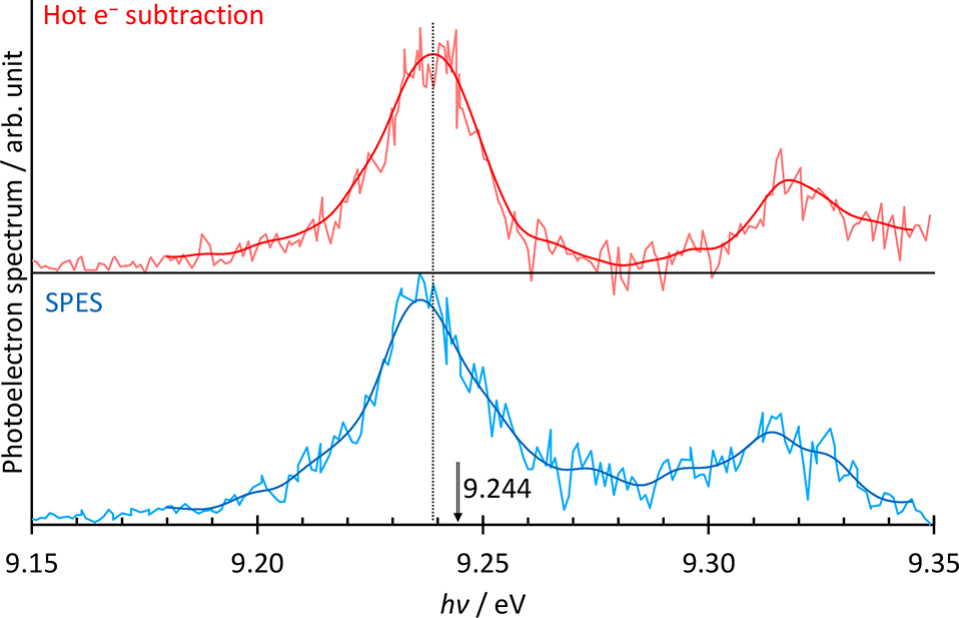
Comparison of hot electron subtracted and slow photoelectron spectra of benzene. The smoothed curves were obtained by applying a 3.3 meV^–1^ low-pass filter. The known ionization energy of benzene is shown as 9.244 eV.

Reconstructing the electron VMI and plotting the spectrum based on data recorded at a single photon energy is best for analyzing angular anisotropies but results in an energy-dependent resolution in the spectrum.^64^ The (ms-)SPES, constructed by inverting the electron VMI and taking only slow, e.g., <40 meV kinetic energy,^65^ electrons into account, promises better S/N ratios at essentially the same resolution as by discriminating for threshold electrons.^33^ Some might argue that signal levels have to be high enough for the electron VMI to be invertible, which already allows one to plot the hot electron subtracted TPES. As shown in Figure 6, this appears not to be the case. Here, we evaluated the first 0.5 s worth of data from the 30 s benzene scan points, which never contained more than 200 threshold electron counts. The spectra were evaluated by hot electron subtraction at a 4 meV electron cutoff energy as well as by SPES based on less than 40 meV kinetic energy electrons. Also shown are two traces obtained by applying a 3.3 meV^–1^ low-pass filter to smooth the spectra. The root-mean-square deviation from the respective smoothed curved is 1.3 times larger for the TPES than for the SPES, confirming that the SPES S/N ratio is indeed superior even at very low signal levels. It is nevertheless surprising that, although the falling edge of the peak is not affected by the subtraction issues in the SPES, the peak maximum is only red-shifted to 9.236 eV from 9.244 eV, which is somewhat less than the 10 meV Stark shift. The momentum and, thus, energy calibration of the electron VMI was based on argon images. When we changed the kinetic energy calibration by ±5%, the peak maximum shifted slightly by 1 meV. Thus, part of the 2 meV discrepancy between the expected, Stark-shifted maximum at 9.234 and the observed 9.236 eV may be due to nonlinearities in the momentum calibration of the detector. Thus, the price to pay for the better SPES S/N ratio is the need to symmetrize and invert the electron images, which may also represent a minor source of uncertainty along the energy axis, similar to the uncertainties encountered in the hot electron subtraction. However, as shown in this discussion also in section 4.2, these effects play only a minor role and may only need to be considered when making precision measurements for determining accurate adiabatic ionization energies to evaluate theoretical methods or when close-lying ionization energies of rotamers and diastereomers complicate isomer-selective assignment in catalysis experiments.

## 5 Database: Photoelectron Photoion Spectral Compendium

**Figure 7:**
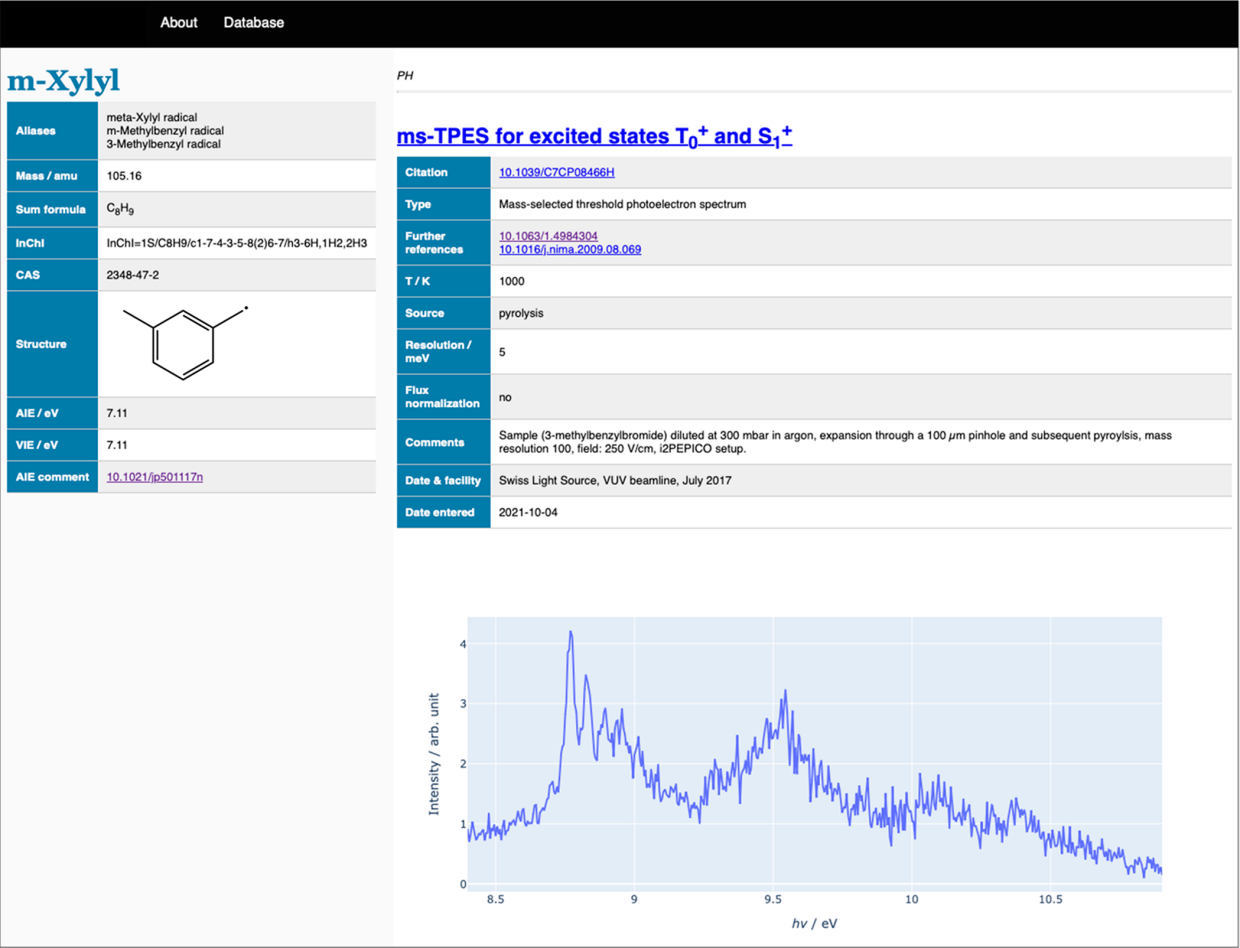
Screenshot of the PhotoElectron PhotoIon Spectral COmpendium hosted at PSI. https://pepisco.psi.ch.

Photoelectron spectra of stable and transient species have been collected and published previously, notably by Kimura,^66,67^ Turner,^68^ Dyke,^15^ and Cockett et al.^69^ While such compilations make it considerably easier to narrow and identify the spectral carriers of ms-TPES, one is often faced with the need to record reference spectra of relatively small and ubiquitous species. These are often published as Supporting Information, fade into obscurity, and must be rerecorded repeatedly. Furthermore, 17 and 40 isomer structures were considered to identify the 129 and 130 amu products of the phenyl + acrylonitrile addition–elimination reaction, respectively.^70^ Franck–Condon simulations could also be useful in future investigations, but the isomer exploration and the simulations would likely have to be repeated. While photoionization cross sections have been compiled by the Hefei group,^71^ the lack of a general photoelectron and photoion spectral compendium complicates the assignment of threshold photoionization and photoionization matrices more with each passing year.^5,6,72^ To make spectral assignment easier and, possibly, automatic in the future, we have established an online PhotoElectron PhotoIon Spectral Compendium at https://pepisco.psi.ch (see Figure 7), with an initial data set of 140 spectra on 60 species.

We solicit experimental data, be it threshold or slow photoelectron spectra, photoion mass-selected or not, photoionization spectra, and Franck–Condon simulations to expand this compendium and support PIMS and PEPICO data analysis of complex reactive mixtures. In addition, the database contains information on how the data were taken, summarizing experimental conditions, including fields, resolution, reactor temperatures, and flow rates as well as computed adiabatic ionization energies. These metadata will establish the context of the spectra and help experimentalists to reliably interpret ms-TPES, ms-SPES, or PI spectra isomer-selectively.

The PEPISCO database will provide a starting point for new users to become familiar with synchrotron-based photoionization techniques and to understand their opportunities and limits. Furthermore, knowledge gained over the last decades must be shared and maintained in a sustainable way to serve an increasingly growing community using PI and PES methods at synchrotron facilities not only in heterogeneous catalysis, combustion, and kinetics but also in the astrochemical context. In the context of novel artificial intelligence approaches in chemistry, our database has the potential to expand to include a large number of spectra and ionization energies, enabling automatic isomer-specific assignment in the future.

## 6 Conclusions and Perspective

We discuss photoion mass-selected threshold photoelectron spectroscopy (ms-TPES), a powerful isomer-selective detection tool in catalysis. The methodology, which combines mass spectrometry and threshold photoelectron spectroscopy by imaging photoelectron photoion coincidence, provides high sensitivity and (isomer) specificity for real-time monitoring of reactants, reactive intermediates, and products. Ionization energies, Franck–Condon simulations, and literature reference spectra identify the vibrational progressions in the ms-TPE spectra to assign neutral spectral carriers.

The benefits of ms-TPES detection combined with cation velocity map imaging are illustrated by their application in studying the catalytic pyrolysis of lignin model compounds, the methanol-to-olefin process, and propane oxybromination. This Perspective also addresses the robustness of ms-TPE spectroscopy, where we focus on temperature and sampling effects and their influence on isomer-selective assignments. It is in this context that ion velocity map imaging (VMI) is demonstrated as a valuable tool to distinguish the molecular beam signal due to hot samples emanating directly from the reactor and the rethermalized background signal in the ionization chamber.

Hot electron subtraction and slow photoelectron spectroscopy are presented as alternative VMI data analysis approaches. While the latter offers better S/N ratios, the former is conceptually simpler to apply in real time. The electron cutoff energy in hot electron subtraction and the VMI energy calibration in SPES can affect peak widths and shift maxima in the photoelectron spectrum. However, these effects, together with the Stark shift, are shown to be minor and only need to be accounted for in high-accuracy measurements when close-lying ionization energies of rotamers and diastereomers are targeted.

In the future, the ms-TPES technique will likely play an increasing role in the advancement of catalysis research and has potential to be expanded from the gas phase also to the liquid phase.^73−75^ The PEPISCO database of photoelectron and photoionization spectra at the Paul Scherrer Institute, along with the availability of literature sources for photoelectron spectra, will further facilitate its application in analytical studies. As the technique continues to evolve, it is anticipated that ms-TPES will provide valuable insights into the mechanistic and kinetic aspects of catalytic processes, ultimately contributing to the development of more efficient and sustainable catalytic systems. We hope that future users of the PEPICO and ms-TPES technique welcome this perspective as a valuable source.
